# Phasing nanopore genome assembly by integrating heterozygous variations and Hi-C data

**DOI:** 10.1093/bioinformatics/btae712

**Published:** 2024-11-27

**Authors:** Jun Zhang, Fan Nie, Feng Luo, Jianxin Wang

**Affiliations:** School of Computer Science and Engineering, Central South University, Changsha, Hunan 410083, China; Xiangjiang Laboratory, Changsha, Hunan 410205, China; Hunan Provincial Key Lab on Bioinformatics, Central South University, Changsha, Hunan 410083, China; School of Computer Science and Engineering, Central South University, Changsha, Hunan 410083, China; Xiangjiang Laboratory, Changsha, Hunan 410205, China; Hunan Provincial Key Lab on Bioinformatics, Central South University, Changsha, Hunan 410083, China; School of Computing, Clemson University, Clemson, SC 29634-0974, United States; School of Computer Science and Engineering, Central South University, Changsha, Hunan 410083, China; Xiangjiang Laboratory, Changsha, Hunan 410205, China; Hunan Provincial Key Lab on Bioinformatics, Central South University, Changsha, Hunan 410083, China

## Abstract

**Motivation:**

Haplotype-resolved genome assemblies serve as vital resources in various research domains, including genomics, medicine, and pangenomics. Algorithms employing Hi-C data to generate haplotype-resolved assemblies are particularly advantageous due to its ready availability. Existing methods primarily depend on mapping quality to filter out uninformative Hi-C alignments which may be susceptible to sequencing errors. Setting a high mapping quality threshold filters out numerous informative Hi-C alignments, whereas a low mapping quality threshold compromises the accuracy of Hi-C alignments. Maintaining high accuracy while retaining a maximum number of Hi-C alignments can be challenging.

**Results:**

In our experiments, heterozygous variations play an important role in filtering uninformative Hi-C alignments. Here, we introduce Diphase, a novel phasing tool that harnesses heterozygous variations to accurately identify the informative Hi-C alignments for phasing and to extend primary/alternate assemblies. Diphase leverages mapping quality and heterozygous variations to filter uninformative Hi-C alignments, thereby enhancing the accuracy of phasing and the detection of switches. To validate its performance, we conducted a comparative analysis of Diphase, FALCON-Phase, and GFAse on various human datasets. The results demonstrate that Diphase achieves a longer phased block N50 and exhibits higher phasing accuracy while maintaining a lower hamming error rate.

**Availability and implementation:**

The source code of Diphase is available at https://github.com/zhangjuncsu/Diphase

## 1 Introduction

Haplotype-resolved genome assemblies hold significant potential for advancing various fundamental research fields. Recent advancement in long-read sequencing technologies have led to the creation of numerous high-quality *de novo* genome assemblies (Logsdon *et al.* 2020, Rhie *et al.* 2021). Notably, the Human Pangenome Reference Consortium has recently unveiled the initial draft of the human pangenome reference, comprising 47 phased diploid assemblies (Liao *et al.* 2023). Despite these achievements, many long-read genome assemblers tend to collapse different haplotypes into a single consensus assembly without additional sequencing data. Unfortunately, this amalgamation results in mosaic haplotypes that incorporate falsely linked variants absent in either haplotype, thereby undermining the integrity of biological inference. Some assemblers have sought to address this issue by leveraging heterozygous variations between haplotypes to maintain local phasing (Chin *et al.* 2016, Cheng *et al.* 2021). However, these assemblers often produce phased blocks of limited length due to constraints imposed by low heterozygosity samples. To attain longer contigs, these assemblers concatenate these short-phased blocks, resulting in a primary assembly. Nonetheless, haplotypes within each contig may switch randomly. The remaining blocks, also known as haplotigs, are relegated to an alternate assembly.

Read-based phasing methods like Whatshap (Martin *et al.* 2016) and HapCut2 (Edge *et al.* 2017) hinge on an established reference. First, reads are mapped to the reference, after which a collection of potential sites, encompassing single nucleotide variants and insertions and deletions, are identified based on the mappings. Phasing of the reads follows by identifying a partition that optimizes the agreement among reads encompassing the different alleles. However, read-based phasing can be affected by reference bias (Günther and Nettelblad 2019), resulting in relatively brief phased blocks.

To generate haplotype-resolved genome assemblies from diploid samples, various tools require additional sequencing data, such as parental short-read data, strand sequencing data, or Hi-C data. Trio-binning approaches like TrioCanu (Koren *et al.* 2018) and hifiasm (trio) (Cheng *et al.* 2022) leverage short-read sequencing data from the parents to identify parent-specific k-mers. Using these k-mers, long-read sequence data from the offspring can be categorized into paternal and maternal bins. While the phased assemblies generated by trio binning are accurate, their practical utility is constrained by the necessity of parental sequencing data, which may not always be accessible. In response to this limitation, single-sample haplotype-resolved assembly methods employing additional strand sequencing (strand-seq) or Hi-C data have emerged (Garg *et al.* 2021, Kronenberg *et al.* 2021, Porubsky *et al.* 2021, Cheng *et al.* 2022, Lorig-Roach *et al.* 2023). Strand-seq-based approaches retain the structural continuity of individual homologs. However, the requirement for living cells and one round of cell division poses challenges when scaling up for multiple species or individuals within a species. On the other hand, proximity ligation data types like Hi-C offer extensive contact information for phasing across longer genomic ranges and can be sequenced from the same sample. Hi-C data proves to be a more versatile and generic solution for phasing. Hifiasm (hic) leverages both HiFi and Hi-C data to produce haplotype-resolved assemblies. While the outcomes achieved by hifiasm (hic) boast remarkable accuracy, its integration within hifiasm restricts its applicability solely to processing HiFi data, thereby constraining its utility for Nanopore data analysis.

Utilizing Hi-C data to phase and extend primary and alternate assemblies into two homologous haplotypes is an effective solution, especially when dealing with pseudo-haplotype diploid assemblies generated by many assemblers. FALCON-Phase is designed to work with primary/alternate assembly format, leveraging Hi-C data for phasing and extension. However, it skips SNP calling, which can compromise the overall accuracy. The method applies mapping quality and edit distance as filters to exclude uninformative Hi-C alignments. Unfortunately, this filtering process leads to the exclusion of numerous informative Hi-C alignments, causing a notable reduction in the overall number of Hi-C alignments and subsequently compromising phasing accuracy, especially in scenarios with high sequencing errors, such as Nanopore sequencing data. The count of Hi-C links experiences a substantial decrease as the mapping quality threshold increases. Moreover, FALCON-Phase lacks a mechanism for handling existing switches within blocks, further undermining its phasing performance. During phasing, FALCON-Phase groups blocks along each primary contig and processes each group separately. For a given block, FALCON-Phase relies solely on the phasing information and Hi-C contacts in front of it within the group. This approach leads to a loss of valuable phasing information and Hi-C contacts behind the block, ultimately impacting the overall phasing accuracy.

To tackle these challenges, we introduce Diphase, an innovative phasing pipeline that leverages heterozygous variations and fully utilizes Hi-C contact information. The workflow begins by mapping raw reads to the primary and alternate assembly, followed by SNP calling using Clair3 (Zheng *et al.* 2022). Uninformative Hi-C alignments are meticulously filtered out, considering mapping quality, edit distance, and the identified SNPs. Diphase incorporates a mechanism that determines mis-assembly used in SALSA2 (Ghurye *et al.* 2019) to detect switches based on the coverage of these filtered Hi-C alignments. Additionally, Diphase generates Hi-C contacts using the filtered Hi-C alignments and phases the blocks using all available Hi-C contacts within each group. Diphase operates on primary/alternate assembly format. In comparative evaluations with FALCON-Phase and GFAse (Lorig-Roach *et al.* 2023) across diverse human datasets, Diphase consistently demonstrates superior performance. It achieves a longer phased block N50 compared to FALCON-Phase and GFAse, while also exhibiting higher phasing accuracy and a lower hamming error rate.

## 2 Materials and methods

**Figure 1.**
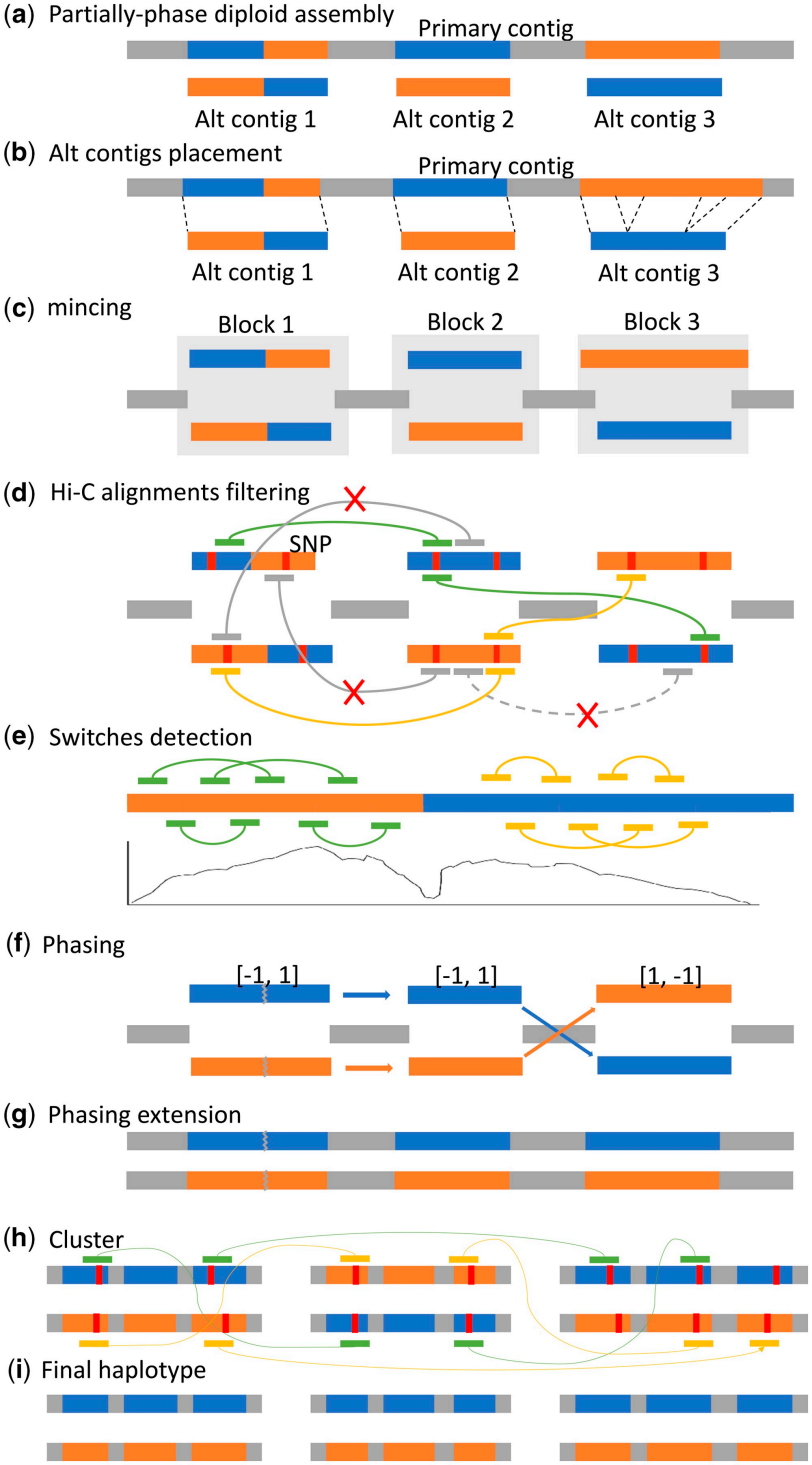
Pipeline of Diphase. (a) Pseudo-haplotype diploid assembly comprises of primary contigs and alternate contigs. Alternate contigs may contain two haplotype sub-blocks (alternate contig 1). (b) Alternate contigs are aligned to its associated primary contigs, an alternate contig may have multiple aligned segments (alternate contig 3). (c) The aligned information is used to mince the primary contigs. Mincing defines the blocks and collapsed haplotypes. (d) SNPs are used to filter the Hi-C alignments. The Hi-C mate-pair is filtered out if either read does not cover at least one SNP. (e) Switches are detected by the coverage of the filtered Hi-C alignments and the blocks are minced by the switch points determined by the detection algorithm. (f) Blocks are assigned to 1 or −1 with the phasing algorithm. (g) The output of Diphase is two full-length haplotypes. (h) Utilizing the filtered Hi-C alignments, cluster paternal and maternal contigs into the same group. (i) Final haplotype.

Beginning with a primary and alternate assembly as the input (Fig. 1a), Diphase phases and extends the contigs using raw reads and Hi-C data from the same sample, ultimately producing two distinct phased haplotypes. The method makes effective use of SNPs identified from raw reads to filter out uninformative Hi-C alignments, subsequently detecting switches in blocks and reorganizing these blocks into haplotypes along a given primary contig. Alternate contigs are aligned to their corresponding primary contigs to delineate their respective haplotype regions (Fig. 1b). Breakpoints, referred to as “minces,” are introduced to separate heterozygous regions from collapsed regions along the primary contigs (Fig. 1c). SNPs identified from raw read alignments to the partially phased diploid assembly play a pivotal role in filtering out uninformative Hi-C alignments (Fig. 1d). The filtered Hi-C alignments are then utilized to calculate coverage, aiding in the detection of switches within the block (Fig. 1e). Hi-C contacts, quantified through the filtered Hi-C alignments, serve as the basis for categorizing haplotype blocks that share the same phase along each contig (Fig. 1f). The collapsed sequences are thoughtfully integrated into both haplotypes to expand the assembly sequences, resulting in two haplotypes encompassing either paternal or maternal blocks, along with the collapsed regions (Fig. 1g).

### 2.1 Datasets

Our experiments were conducted on five human datasets, namely NA12878, HG00733, HG002, HG002 simplx, and HG002 duplex. The datasets for NA12878, HG00733, and HG002 were sequenced using R9 chemistry, while the datasets for HG002 simplex and HG002 duplex were sequenced using R10 chemistry. R10 chemistry has higher accuracy compared to R9. The five datasets along with their respective Hi-C data, were downloaded to assess the performance of Diphase. Restriction enzymes GATC and GANTC are employed to generate the Hi-C data for NA12878. For HG00733 and HG002, a combination of restriction enzymes is used, including GATC, GANTC, CTNAG, and TTAA. We used the primary/alternate assembly format of PECAT (Nie *et al.* 2022) on the five datasets. We utilized Shasta (Shafin *et al.* 2020) to assemble the three datasets of HG002 for performance comparison with GFAse The details of the five datasets are shown in Table 1. All the data used in our experiments are publicly accessible, and the sources are provided in Supplementary Table S1. All the configuration used for the assemblies are listed in Supplementary Note S3.

**Table 1.** Details of data used in evaluation.

Dataset	Basecaller	Coverage	Match (%)	Mismatch (%)	Insertion (%)	Deletion (%)	N50 (kbp)	Hi-C coverage
NA12878	Guppy 5	48	92.10	2.28	1.90	3.73	15	44
HG00733	Guppy 4	82	91.05	2.81	2.42	3.73	30	40
HG002	Guppy 6	59	94.78	1.88	1.27	2.07	80	73
Simplx	Guppy 6	52	98.77	0.41	0.32	0.50	32	73
Duplex	Dorado 0.1.1	45	99.65	0.08	0.09	0.77	35	73

Coverage is calculated as the number of total bases divided by genome size, where the genome size is set to 3 Gb for the five datasets. “match,” “mismatch,” “insertion,” and “deletion” are the percentages of matches, mismatches, insertions, and deletions in the alignments between the reads and the reference genome.

### 2.2 Alternate contigs placement

With the input of primary contigs and alternate contigs (Fig. 1a), alternate contigs are aligned to their respective primary contigs (as shown in Fig. 1b) using minimap2 v2.22 (Li 2021). In cases where an alternate contig exhibits multiple aligned segments, Diphase employs a dynamic programming algorithm to optimally chain these segments, thereby maximizing the alignment. For a given list of aligned segments associated with an alternate contig, they are sorted by their starting positions on the primary contig. The maximal alignment length up to the *i*-th aligned segment, denoted as *f*(*i*), is calculated using the following formula:

(1) $$f\left(i\right)=\max\left\{\underset{i>j\geq 1}{\max}\left\{f\left(j\right)+\left|S_{i}\right|-\text{gap}\left(j,i\right)\right\},\left|S_{i}\right|\right\}$$

Here, $\left|S_{i}\right|$ represents the alignment length of the *i*-th segment, and gap(*j*, *i*) denotes the distance on the primary contig between the end of the *j*-th segment and the start of the *i*-th segment. If both segments overlap, gap(*j*, *i*) sets to 0. This process continues until all aligned segments have been traversed, yielding the maximal *f*(*i*). Subsequently, backtracking is applied to determine the best chain. *P*(*i*) represents the index of the optimal predecessor of segment *i*. If $f(i)=|S_{i}|$, *P*(*i*) is set to 0. Otherwise, it is determined as $\arg\max\left\{f\left(j\right)+\left|S_{i}\right|-\text{gap}\left(j,i\right)\right\}$. For the segment *i* with the highest *f*(*i*), the backtracking procedure is repeated until *P*(*i*) reaches 0. This results in the identification of the best chain, with the aligned segments within this chain retained, while others are filtered out. Diphase preserves one of the identical alignments and filters out those alignments fully contained by others (Algorithm 1 in Supplementary Materials). This process generates three separated minced FASTA files, each providing information on the placement of alternate contigs: *hap*1, *hap*2, and *collapsed* (depicted in Fig. 1c). *Hap*1 encompasses the filtered alternate contigs, *hap*2 comprises the corresponding haplotype regions along the primary contigs, and *collapsed* represents the collapsed region of the assembly. Additionally, pairing information between *hap*1 and *hap*2 is generated during this process.

### 2.3 Hi-C alignments filtering

Raw reads undergo mapping to primary contigs and alternate contigs using minimap2 v2.22. Subsequently, Clair3 (Zheng *et al.* 2022) is employed to call SNPs on primary contigs and alternate contigs, leveraging separate alignments for each. The mappings of each position between alternate contigs and primary contigs are meticulously recorded in the memory by parsing the alignment records of both alternate contigs and primary contigs. Suppose *B_a_* represents the nucleotide base at position *x_a_* on an alternate contig, *x_p_* signifies its mapped position on the associated primary contigs, and *B_p_* denotes the nucleotide base at position *x_p_*. The alternate bases called by Clair3 at positions *x_p_* and *x_a_* are denoted as ALT_*p*_ and ALT_*a*_, respectively. When ALT_*p*_ = *B_a_* and ALT_*a*_ = *B_p_*, the SNPs at positions *x_p_* and *x_a_* are utilized to filter Hi-C alignments, while the remaining SNPs are discarded. This check ensures the accuracy of the SNPs, contributing to the refinement of the overall process.

Upon merging the *hap*1, *hap*2, and *collapsed* FASTA files into a single minced file, Diphase proceeds by mapping the Hi-C reads to the minced contigs using BWA-MEM (Li 2013). The resulting alignments are directed to SAMtools (Li *et al.* 2009) for further processing, removing unmapped, secondary, and supplementary alignments (with parameter −F2316). This step ensures that each read in the mate-pair has only one alignment record, improving data integrity. Another critical filtration step is applied to the Hi-C alignments (as depicted in Fig. 1d). Alignments satisfying the following conditions remain unfiltered: (i) having a mapping quality score greater than a specified threshold, *q* (default 10), and an edit distance less than a threshold, *e* (default 5); or (ii) covering at least one SNP retained in the previous step. We adopt the same thresholds for mapping quality score and edit distance as those used in FALCON-Phase. Alignments that fail to meet these criteria are filtered out. If both alignments in a mate-pair successfully pass the filter, these alignments are utilized for the detection of switches and phasing. Conversely, mate-pair alignments that do not meet the filter criteria are excluded from further analysis (Algorithm 2 in Supplementary Materials).

### 2.4 Switch detection

Ideally, an individual alternate contig represents either a paternal haplotig or a maternal haplotig. However, certain segments of the alternate contigs may contain both paternal sub-blocks and maternal sub-blocks (as illustrated in Fig. 1e) due to the erroneous phasing by the assemblers. Notably, cis-interactions for Hi-C mate-pairs tend to outweigh trans-interactions. Consequently, if a switch occurs within a contig, the interactions for Hi-C mate-pairs between the two sub-blocks decrease significantly compared to the interactions within each sub-block (Fig. 1e), resulting in a pronounced decline in coverage, as calculated by the filtered Hi-C alignments. The coverage of a Hi-C mate-pair is defined as the region on the contig spanning from the start position of the leftmost fragment to the end position of the rightmost fragment. The mis-assembly detection algorithm implemented in SALSA2 (Ghurye *et al.* 2019) is applied because the switch is likely a mis-assembly. The algorithm employs a range of thresholds to detect a group of suspicious intervals, pinpointing continuous regions with lower coverage. In cases where multiple intervals are detected for a specific threshold, the interval with the maximum size is selected. These intervals signify regions on the contig that may potentially contain switch points. The maximal set of intervals intersecting at any location along the contig signifies the region with consistently low coverage across the tested cutoffs, indicating the presence of a switch point. During this process, any detected switch on a block will be filtered if no switch is identified on its corresponding haplotype block (Algorithm 3 in Supplementary Materials), thereby reducing the occurrence of false positives. To further minimize false positives, additional checks are conducted after the phases of the blocks are determined. Diphase utilizes phase information to reposition blocks while considering existing switches. Switches within a block are disregarded if the sub-blocks have the same phase.

### 2.5 Phasing

The number of Hi-C mate-pairs linking two haplotigs represents the Hi-C contacts between them in the filtered Hi-C alignments. Let us consider two haplotigs, *a* and *b*, along with their corresponding haplotypes, $a^{\prime }$ and b′. We can measure four contacts: *w_ab_* for *a* and *b*, $w_{ab\prime }$ for *a* and $b\prime ,\;w_{a^{\prime }b}$ for $a^{\prime }$ and *b*, and $w_{a\prime b\prime }$ for a′ and b′. When *a* and *b* originate from the same haplotype, the contacts *w_ab_* and $w_{a\prime b\prime }$ tend to be larger than $w_{ab\prime }$ and $w_{a\prime b}$. Conversely, if *a* and *b* come from different haplotypes, then $w_{ab\prime }$ and $w_{a\prime b}$ are typically larger than *w_ab_* and $w_{a\prime b\prime }$. The primary goal of the phasing algorithm is to maximize the contacts of the same haplotypes. Here, $\theta_{a}\in \left\{1,-1\right\}$ denotes the phase of haplotype *a* (Fig. 1f). We can define the objective function $f(\theta_{a},\theta_{b})$ as:

(2) $$f\left(\theta_{a},\theta_{b}\right)=\{\begin{array}{cc} w_{ab}+w_{a\prime b\prime }-\left(w_{a\prime b}+w_{ab\prime }\right) & \theta_{a}\theta_{b}=1\\ w_{a\prime b}+w_{ab\prime }-\left(w_{ab}+w_{a\prime b\prime }\right) & \theta_{a}\theta_{b}=-1 \end{array}$$

or equivalently

(3) $$f\left(\theta_{a},\theta_{b}\right)=\theta_{a}\theta_{b}\left(w_{ab}+w_{a\prime b\prime }-\left(w_{a\prime b}+w_{ab\prime }\right)\right)$$

where *w_ab_*, $w_{ab\prime },\;w_{a\prime b}$ and $w_{a\prime b\prime }$ can be counted with the filtered Hi-C alignments. *θ_a_* and *θ_b_* are variables whose values will be determined by the phasing algorithm. The composite objective function of $\vec{\theta }$ overall blocks is

(4) $$f\left(\vec{\theta }\right)=\sum_{a,b}\theta_{a}\theta_{b}\left(w_{ab}+w_{a\prime b\prime }-\left(w_{a\prime b}+w_{ab\prime }\right)\right)$$

We utilize a stochastic algorithm (Tourdot *et al.* 2021, Cheng *et al.* 2022) (Algorithm 4 in Supplementary Materials) to solve (4), which represents the composite objective function of all blocks. Once the phases of all the blocks are determined, Diphase subsequently repurposes the blocks to account for existing switches. In cases where the phases of the two sub-blocks are the same, switches within the block are disregarded. The blocks are then combined into two full-length haplotypes, incorporating the collapsed regions based on their determined phases (Fig. 1g). The contigs are subsequently clustered into paternal and maternal haplotypes using the phasing algorithm with filtered Hi-C alignments (Fig. 1h and i).

## 3 Results

### 3.1 Evaluation metrics

We downloaded the parental short-read data to assess the performance of Diphase using Merqury (Rhie *et al.* 2020). A phased block is defined as a group comprising a minimum of two haplotype-specific k-mers (k-mers appearing in exactly one haplotype, but not the other) originating from the same haplotype. The phased block N50 denotes the length of the phased block, encompassing at least 50% of all the phased blocks. The switch error rate is determined by calculating the fraction of haplotype-specific k-mers within a phased block that are assigned to the wrong haplotype. The hamming error rate is computed using a custom script as $\text{min}\left(N_{\text{pat}},N_{\text{mat}}\right)/(N_{\text{pat}}+N_{\text{mat}})$, where *N*_pat_ and *N*_mat_ represent the number of paternal and maternal specific k-mers, respectively. Gene completeness was estimated by BUSCO (Simão *et al.* 2015). Phasing errors are employed to assess the phasing ability of the tools without taking switches within each block into account. Additional details about phasing errors are provided in Supplementary Note S1.

### 3.2 Performance evaluation of Diphase

#### 3.2.1 Phasing performance on PECAT assemblies

**Figure 2.**
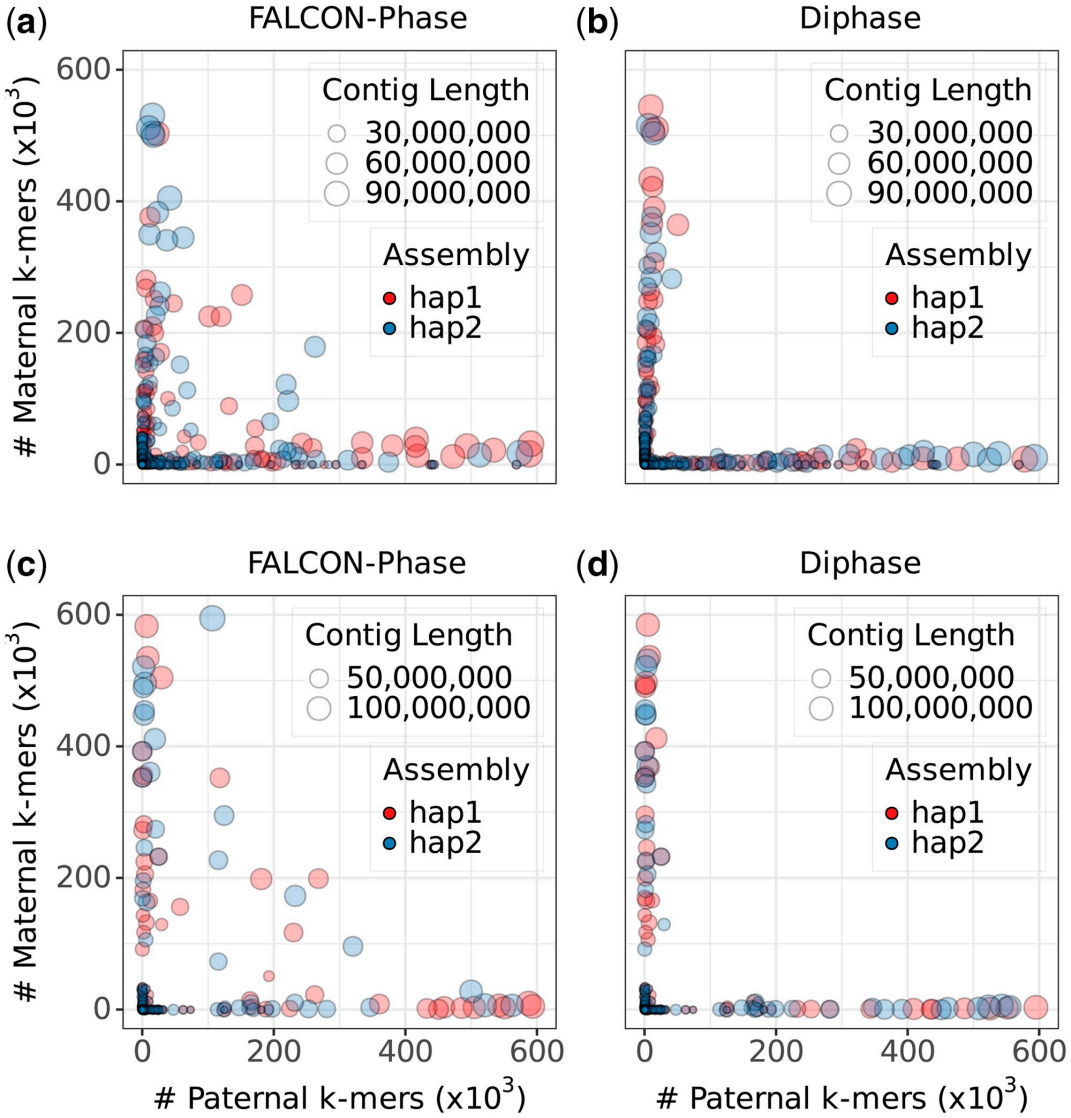
Phasing accuracy of FALCON-phase and Diphase on PECAT assemblies using R10 datasets. Size of the data point indicates the size of the contig. Its coordinates represent the number of paternal-specific and maternal-specific k-mers. The contigs lie along the axes indicating that they are well-phased. The top of the figure shows the phasing performance of different tools on the HG002 simplex dataset (a: FALCON-Phase, b: Diphase), while the bottom depicts the phasing performance of different tools on the HG002 duplex dataset (c: FALCON-Phase, d: Diphase).

We acquired and evaluated five Nanopore datasets (NA12878, HG00733, HG002, HG002 simplx, and HG002 duplex) to appraise the performance of Diphase. Employing PECAT, we assembled the five datasets and executed Diphase and FALCON-Phase on the resultant assemblies. Overall, Diphase demonstrates superior performance relative to FALCON-Phase (Table 2 and Fig. 2). Across all the five datasets, Diphase exhibits significantly lower hamming error rates and phasing error rates. Specifically, Diphase exhibits hamming error rates more than 10 times lower than those of FALCON-Phase on the HG00733 dataset (2.28% and 1.97% compared to 23.55% and 23.41%). While the switch error rates are comparable, Diphase generates longer phased block N50 values. On the dataset of HG002 duplex, the phased block N50s generated by Diphase are more than 50% longer than those generated by FALCON-Phase. The enhanced phased block N50 improvement becomes more evident in scenarios with fewer hamming error rates in alternate contigs (Supplementary Tables S2 and S3). Gene completeness, genome size, and N50 generated by Diphase and FALCON-Phase are comparable. By leveraging SNPs in the filter, Diphase effectively identifies a larger number of informative Hi-C alignments, contributing to enhanced phasing accuracy and longer phased block N50 values. We then ran dipcall (Li *et al.* 2018) on the phased assemblies and used VCFdist (Dunn and Narayanasamy 2023) to evaluate the performance of FALCON-Phase and Diphase. As shown in Supplementary Table S4, Diphase performs better than FALCON-Phase. Specifically, assemblies phased by Diphase have significantly fewer switch errors than those phased by FALCON-Phase, resulting in longer switch NGC50s.

**Table 2.** Performance on PECAT assemblies.

								Gene completeness	
Dataset	Method	Genome size (Gb)	N50 (Mb)	Phased block N50 (Mb)	Switch error (%)	Hamming error (%)	Phasing error (%)	Single (%)	Dup (%)
NA12878	FALCON-Phase	2.882	43.2	1.4	0.66	31.93	35.87	90.8	1.8
		2.887	43.3	1.4	0.66	31.10	34.56	91.1	1.9
	Diphase	2.874	43.2	1.8	0.66	8.34	7.22	90.8	1.7
		2.876	43.2	1.9	0.68	8.06	6.65	91.0	1.7
HG00733	FALCON-Phase	3.005	82.2	2.7	0.52	23.55	34.43	90.4	1.6
		3.007	82.3	2.7	0.53	23.41	34.57	90.7	1.6
	Diphase	3.002	82.3	3.7	0.53	2.28	8.04	90.7	1.6
		3.000	82.2	3.9	0.51	1.97	8.16	90.5	1.6
HG002	FALCON-Phase	3.053	83.1	16.5	0.09	5.53	10.40	91.5	1.7
		3.047	83.3	18.7	0.10	2.85	11.34	91.4	1.8
	Diphase	3.042	83.2	22.0	0.09	0.42	3.88	91.5	1.7
		3.045	83.3	22.0	0.10	0.74	4.34	91.4	1.7
HG002 simplx	FALCON-Phase	2.904	49.9	2.1	0.18	5.64	16.11	93.0	1.7
		2.905	49.8	2.1	0.18	5.93	16.00	93.1	1.6
	Diphase	2.905	49.8	3.1	0.18	2.24	3.06	93.1	1.7
		2.904	49.9	3.1	0.18	2.12	3.04	93.0	1.7
HG002 duplex	FALCON-Phase	3.016	77.2	8.4	0.08	3.72	16.84	90.1	1.7
		3.017	77.2	8.1	0.08	3.21	17.06	90.1	1.7
	Diphase	3.015	77.2	13.7	0.08	0.98	5.24	90.1	1.7
		3.017	77.1	14.2	0.08	0.67	5.64	90.1	1.6

Haplotype 1 is reported on top and haplotype 2 on bottom of each row.

#### 3.2.2 Phasing performance on Shasta assemblies

**Figure 3.**
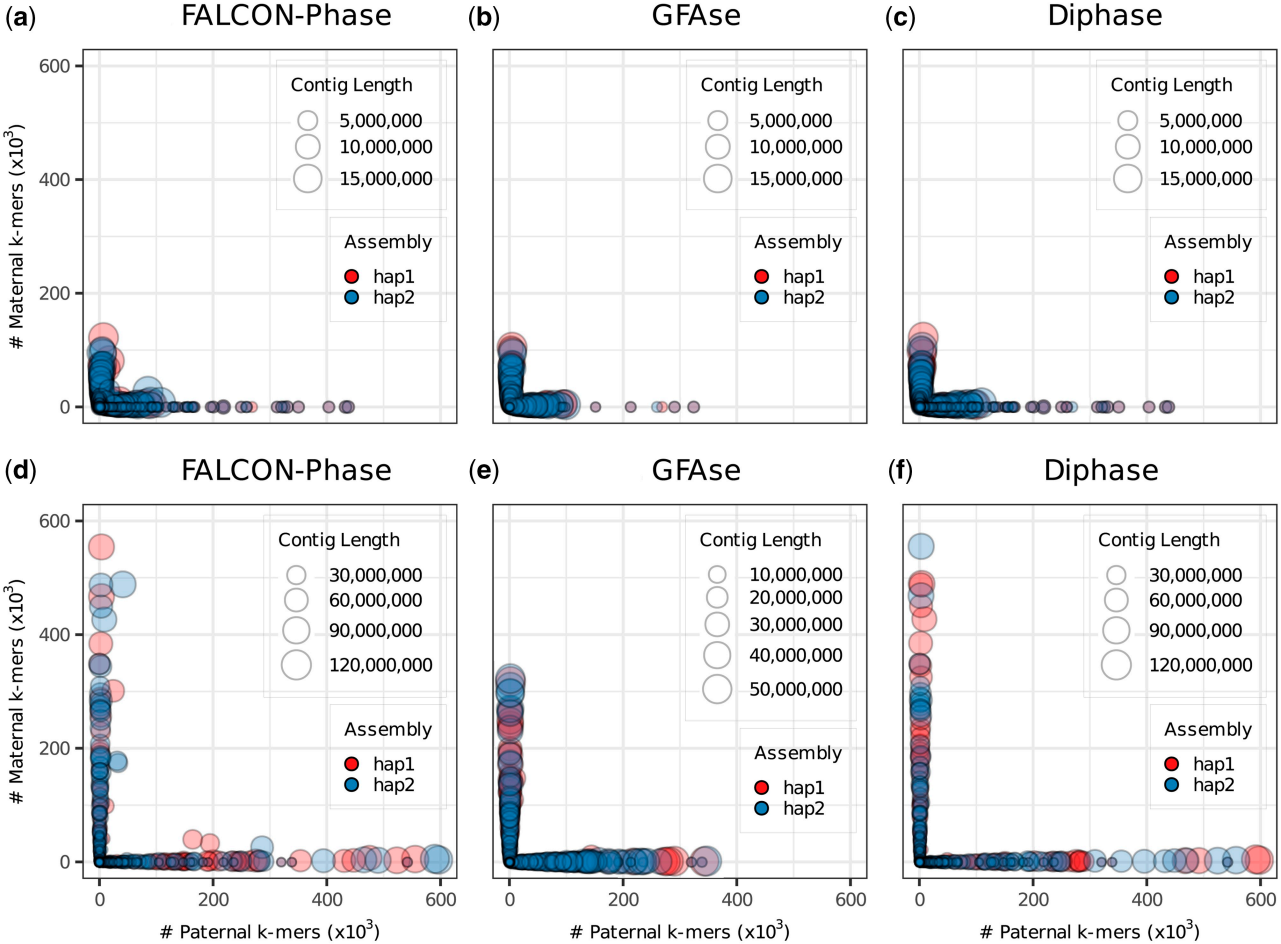
Phasing accuracy of FALCON-Phase, GFAse, and Diphase on Shasta assemblies using R10 datasets. Size of the data point indicates the size of the contig. Its coordinates represent the number of paternal-specific and maternal-specific k-mers. The contigs lie along the axes indicating that they are well-phased. The top of the figure shows the phasing performance of different tools on the HG002 simplex dataset (a: FALCON-Phase, b: GFAse, c: Diphase), while the bottom depicts the phasing performance of different tools on the HG002 duplex dataset (d: FALCON-Phase, e: GFAse, f: Diphase).

We utilized Shasta to assemble the three datasets of HG002, comparing the performance of Diphase with GFAse and FALCON-Phase. The primary/alternate contigs were generated using a python script feeding to FALCON-Phase and Diphase. Additional details on generating inputs for each phasing tool are provided in Supplementary Note S2. As depicted in Table 3 and Fig. 3, Diphase consistently outperform FALCON-Phase, as observed previously. The hamming error and phasing error from Diphase are significantly lower compared to those produced by FALCON-Phase. Additionally, Diphase yields a longer phased block N50 than FALCON-Phase while demonstrating comparable results in switch error and gene completeness. When compared to GFAse, the switch error and hamming error generated by Diphase are smaller than those generated by GFAse. Diphase also produces a longer phased block N50 than GFAse. When evaluated by VCFdist, Diphase performs the best (Supplementary Table S4). Moreover, the gene completeness of the assemblies phased by GFAse is the lowest (Table 3). Specifically, assemblies phased by GFAse contain at least 3% fewer single genes than those phased by the other two tools. The reason is that several input haplotigs remain unphased and are not included in the phased assemblies, whereas FALCON-Phase and Diphase phase all the input haplotigs.

**Table 3.** Performance on Shasta assemblies.

								Gene completeness	
Dataset	Method	Genome size (Gb)	N50 (Mb)	Phased block N50 (Mb)	Switch error (%)	Hamming error (%)	Phasing error (%)	Single (%)	Dup (%)
HG002	FALCON-Phase	3.060	32.8	1.1	3.44	4.53	2.18	93.2	1.6
		3.060	32.8	1.2	3.29	4.25	1.97	93.2	1.6
	GFAse	2.712	26.7	1.1	5.50	6.62	0.75	90.1	1.5
		2.711	26.7	1.1	5.21	6.26	0.56	90.1	1.5
	Diphase	3.059	32.8	1.2	3.28	3.93	0.98	93.2	1.6
		3.061	32.8	1.1	3.42	4.13	0.83	93.2	1.6
HG002 simplx	FALCON-Phase	3.014	3.4	0.6	1.58	5.21	10.85	92.4	1.8
		3.014	3.4	0.6	1.59	5.39	11.53	92.4	1.8
	GFAse	2.636	3.7	0.8	2.31	5.44	2.61	86.9	1.5
		2.636	3.7	0.8	2.35	5.66	2.44	86.9	1.5
	Diphase	3.013	3.4	0.7	1.54	3.82	3.22	92.4	1.8
		3.014	3.4	0.8	1.56	3.82	2.91	92.4	1.8
HG002 duplex	FALCON-Phase	3.100	38.1	4.1	0.26	0.91	5.68	93.8	1.6
		3.099	38.1	4.2	0.25	0.98	5.80	93.8	1.6
	GFAse	2.739	17.6	5.4	0.40	0.72	0.40	90.8	1.5
		2.738	17.5	5.4	0.40	0.68	0.31	90.9	1.5
	Diphase	3.099	38.1	6.2	0.25	0.46	0.68	93.8	1.6
		3.099	38.1	6.7	0.24	0.40	0.43	93.7	1.6

Haplotype 1 is reported on top and haplotype 2 on the bottom of each row.

### 3.3 Performance of the filtering method based on SNP

The exceptional performance of Diphase hinges on its robust filtering method, particularly in scenarios with high sequence error rates. When employing only mapping quality as the filter, the count of Hi-C links diminishes as mapping quality increases. In contrast, by incorporating both mapping quality and heterozygous variations as filters, the decline in the number of Hi-C links occurs more gradually with increasing mapping quality. Heterozygous variations play an important role in the filter. Comparing to using mapping quality only as the filter, the Hi-C links are more accurate if each of the Hi-C mate pair contains at least one heterozygous variation. We conduct an analysis to examine the impact of Diphase’s filtering method on the phasing performance using the five PECAT assemblies. The analysis involves counting the number of Hi-C mate-pairs utilized in the phasing process (Supplementary Table S5), with the exclusion of Hi-C mate-pairs aligned to the same block. A Hi-C mate-pair is considered correct if the two aligned blocks originated from the same haplotype and incorrect otherwise. Leveraging SNP filtering, Diphase retains a significantly higher number of Hi-C mate-pairs for phasing, which also exhibited higher accuracy, ultimately contributing to its superior phasing performance. Notably, in the case of the HG002 dataset, Diphase retains approximately three times more Hi-C mate-pairs compared to FALCON-Phase, and these retained Hi-C mate-pairs demonstrated significantly higher accuracy (75.05% for Diphase compared to 61.96% for FALCON-Phase).

Our analyses extend to evaluating the efficacy of switch detection using the filtered Hi-C alignments. The coverage calculation is based on the two segments in the Hi-C mate-pair, both of which are mapped to the same haplotig. Since the number of haplotigs containing switches is limited, the switches hava a negligible impact on the performance evaluation of the phasing tools. An illustrative example is used to demonstrate Diphase’s capability in switch detection, using the HG002 Nanopore dataset (Supplementary Fig. S1). The example illustrates the haplotig 000023F: 35606921–49533002 and the haplotig 000023F_009:0–13865647 within the same block, exhibiting a switch in the interval [4.6 m, 4.8 m] on 000023F: 35606921–49533002 and [4.6 m, 4.7 m] on 000023F_009:0–13865647, respectively. A discernible drop in the coverage, as calculated by the filtered Hi-C alignments for both haplotigs, is observed around the identified intervals. FALCON-Phase can identify the drop, but it is more apparent when identified by Diphase. The switch points, determined by the detection algorithm, are in close proximity to the intervals for both haplotigs, prompting Diphase to separate the two haplotype sub-blocks based on the detected switch points. The switch detection method in Diphase is designed to identify long-range haplotype block switches, but it cannot detect short switches within a haplotype block. We then evaluated the heterozygous variants affected by switch correction, as shown in Supplementary Table S6. Using switch correction, Diphase identifies more heterozygous variants in PECAT assembly results in the HG002 and simplx datasets. No switches are detected by Diphase in the other four datasets, resulting in identical heterozygous variant counts. We introduced 100 switches using the HG002 dataset assembled by PECAT. Diphase detects 101 switches, of which 98 are simulated, while the remaining 3 are real switches in the assembly.

To assess the influence of Hi-C coverage on the performance of Diphase, we ran Diphase on the Shasta assembly of the HG002 duplex dataset with varying Hi-C coverage (10X, 20X, 30X, 50X, and 100X). The results are shown in Supplementary Fig S2. The performance of Diphase improves with the increasing Hi-C coverage. However, when the coverage exceeds 50X, the improvement becomes less pronounced.

We conducted an experiment where the algorithm determined the optimal alignment from multiple alignments using the Shasta assemblies. When mapping a Hi-C read pair to the contigs, in single alignment mode, only the primary alignment segment (the one with the smallest coordinate) is retained. In contrast, in multiple alignment mode, all mapped segments produced by BWA are saved. In this mode, the most beneficial alignment segment is determined as follows: If the primary alignment segment meets the filtering criteria described in Section “Hi-C alignments filtering,” Diphase designates it as the best alignment segment. If not, Diphase ranks the mapped segments that satisfy the filtering criteria by alignment score and selects the highest-scoring segment as the best alignment. The results are presented in Supplementary Table S7. Diphase’s performance in single and multiple alignment modes is comparable. We opted to use the results from single alignment mode for evaluation due to the simplicity of post-processing. In the future, we will explore more efficient strategies for handling multiple alignment mode to improve performance further.

Diphase requires less time for processing the three HG002 datasets but takes more time for the other two datasets compared to FALCON-Phase (Supplementary Table S8) on PECAT assemblies. When phasing Shasta assemblies, Diphase consumes more time due to the variation calling process.

## 4 Discussion

Haplotype-resolved assembly forms the bedrock of numerous bioinformatics disciplines, including genomics and pangenomics. With the constraints imposed by read length, current long-read assemblers fail to produce haplotype-resolved assemblies without additional data, such as parental short-read data, strand sequencing data, or Hi-C data. Hi-C data, sequenced from the same sample, is particularly suited for phasing. However, existing assemblers and phasing tools utilizing Hi-C data tend to filter out numerous informative Hi-C alignments by relying solely on mapping quality or unique k-mers. To address this, we introduce Diphase, a phasing tool that leverages heterozygous variation to filter out uninformative Hi-C alignments and detect switches in haplotigs. Evaluation of Diphase on several Human datasets demonstrates its superior phasing accuracy compared to FALCON-Phase and GFAse. The evaluation of phasing tools’ performance is affected by switch errors in the haplotigs. To minimize the impact of switch errors, we introduced a new metric, the phasing error rate. The results demonstrate that Diphase successfully achieves a substantial reduction in the phasing error rate.

Diphase currently requires a substantial amount of time for execution due to the use of BWA-MEM for Hi-C mapping and Clair3 for SNP calling. However, if the quality of the raw assemblies is high, we can potentially utilize the differences between the heterozygous regions as SNPs, thus eliminating the need for Clair3. Presently, Diphase operates with the primary/alternate assembly format and Shasta output, which led us to compare it with FALCON-Phase and GFAse, as FALCON-Phase supports the primary/alternate assembly format and GFAse operates with assembly graph. Phasing tools unable to function with those formats were not considered in the comparison. GreenHill (Ouchi *et al.* 2023) is an excellent phasing tool that produces chromosome-level scaffolds. As it does not perform individual contig phasing, we have excluded GreenHill from our comparison. We are currently in the process of developing the code to enable Diphase to work with the dual assembly format and assembly graph.

## Supplementary Material

btae712_Supplementary_Data

## Data Availability

All sequencing data used in this study is publicly available, with download links provided in Supplementary Table S1.
