# Supplementary material for: Phasing nanopore genome assembly by integrating heterozygous variations and Hi-C data
Source: Bioinformatics. 2024 Nov 27;40(12):btae712. doi: 10.1093/bioinformatics/btae712 (PMC11663803; doi:10.1093/bioinformatics/btae712)
Supplement: btae712_Supplementary_Data [file btae712_supplementary_data.pdf]

supplementary information

Phasing Nanopore genome assembly by integrating heterozygous variations and Hi-C data

Jun Zhang[1,2], Fan Nie[1,2], Feng Luo[3] and Jianxin Wang*[1,2,]

[1]School of Computer Science and Engineering, Central South University, Changsha, 410083, Hunan, China
[2]Hunan Provincial Key Lab on Bioinformatics, Central South University, Changsha, 410083, Hunan, China
[3]School of Computing, Clemson University, Clemson, 29634-0974, SC, USA
Jianxin Wang: jxwang@mail.csu.edu.cn

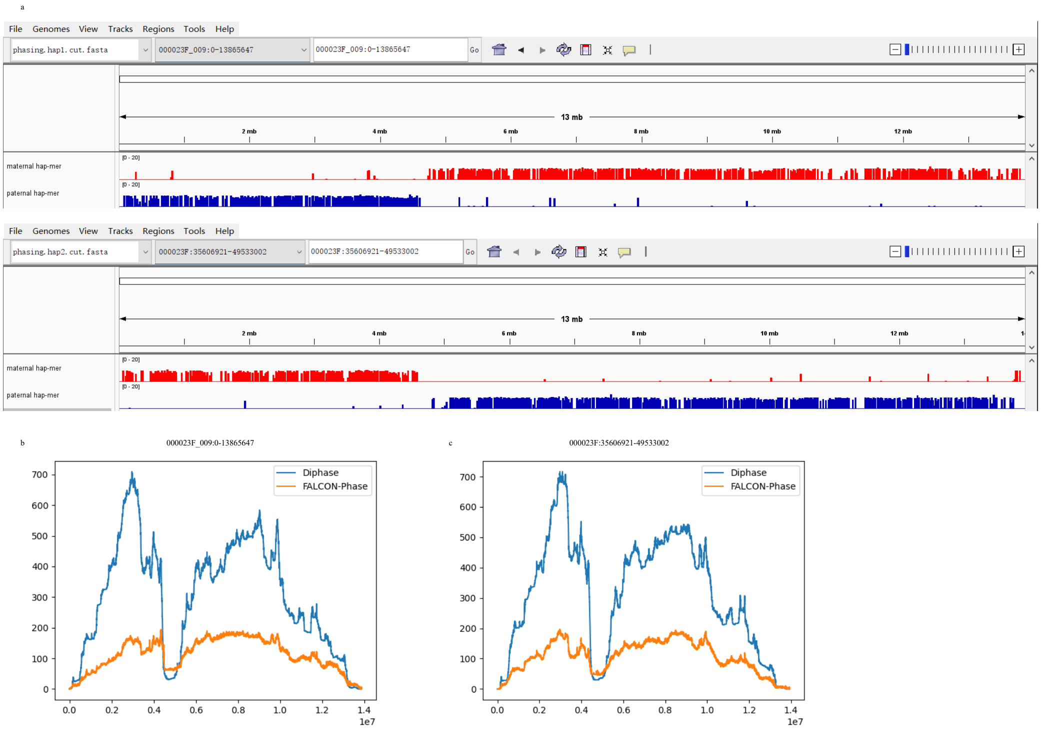

Supplementary Fig S1: **a**. Hap-mers tracks provide by Merqury. **b**. Coverage plots for haplotig 000023F_009:0-13865647. c. Coverage plots for haplotig 000023F:35606921-49533002

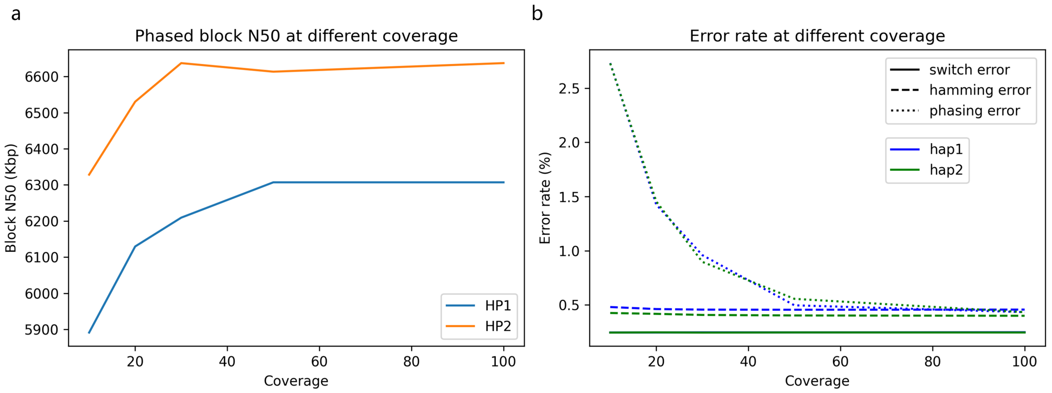

Supplementary Fig S2: We ran Diphase on the Shasta assembly of the HG002 duplex dataset using Hi-C data downloaded from UCSC with the ”HiC_1” files. BWA was used to map the Hi-C data to the assembly. SAMtools view command was employed to generate different coverage levels (10X, 20X, 30X, 50X, and 100X) for the mapping results. **a**. Phased block N50 at different Hi-C coverages. **b**. Switch error rate (solid line), hamming error rate (dashed line) and phasing error rate (dotted line) at different Hi-C coverage

Supplementary Table S1: Detail information of the datasets

datasets	raw reads	Hi-C data	paternal data
NA12878	rel 7	SRR6675327	Merqury
HG00733	HG00733	SRR11347815	PRJNA42573
HG002	HG002	HG002 Hi-C	father, mother
HG002 simplx	HG002 simplx	HG002 Hi-C	father, mother
HG002 duplex	HG002 duplex	HG002 Hi-C	father, mother

Supplementary Table S2: Statistics of the PECAT assemblies

datasets	genome size (Gb)	N50 (Mb)	block N50 (Mb)	switch error (%)	hamming error (%)
NA12878	2.858	43.1	1.4	0.63	39.67
	2.361	1.2	0.9	0.51	4.11
HG00733	2.956	81.0	2.7	0.47	37.55
	2.796	2.4	1.8	0.51	1.48
HG002	3.003	81.8	12.0	0.09	21.24
	2.874	7.3	7.1	0.10	0.54
HG002 simplx	2.869	48.9	1.0	0.12	26.17
	2.220	0.5	0.5	0.05	0.93
HG002 duplex	2.961	75.6	2.7	0.06	24.44
	2.620	1.2	1.2	0.06	0.24

Primary assembly is reported on top and alternate assembly on the buttom of each row.

Supplementary Table S3: Statistics of the Shasta assemblies

datasets	genome size (Gb)	N50 (Mb)	block N50 (Mb)	switch error (%)	hamming error (%)
HG002	3.060	32.8	1.0	3.32	16.68
	2.502	3.9	1.0	5.67	6.61
HG002 simplx	3.014	3.4	0.4	1.45	19.08
	1.709	0.3	0.3	1.81	2.99
HG002 duplex	3.100	38.1	2.2	0.23	21.22
	2.197	1.2	1.2	0.18	0.22

Primary assembly is reported on top and alternate assembly on the buttom of each row.

Supplementary Table S4: Evaluation of phased assemblies

dataset	assembly	method	switch error	flip error	NG50	switch NGC50	switchflip NGC50
HG002	PECAT	FALCON-Phase	630	17,861	138,320,722	12,192,788	282,784
		Diphase	560	17,874	138,320,722	14,932,317	268,551
	Shasta	FALCON-Phase	328	901	144,855,731	37,251,570	5,799,504
		GFAse	217	903	144,851,610	96,181,259	6,586,850
		Diphase	220	902	144,855,731	64,220,680	5,765,512
simplx	PECAT	FALCON-Phase	2,031	9,772	155,803,566	4,102,448	488,545
		Diphase	676	9,846	155,803,566	14,911,038	510,116
	Shasta	FALCON-Phase	1,868	243	155,620,174	3,790,197	3,091,819
		GFAse	161	345	155,620,174	89,817,968	16,587,497
		Diphase	88	245	155,620,174	78,133,974	16,842,085
duplex	PECAT	FALCON-Phase	1,017	7,080	144,989,535	9,582,606	568,631
		Diphase	395	7,118	144,989,535	28,964,641	729,629
	Shasta	FALCON-Phase	369	381	155,748,524	21,854,873	8,250,110
		GFAse	92	447	144,876,089	86,922,666	9,785,619
		Diphase	57	389	155,748,524	91,998,343	10,680,030

We used dipcall (v0.3) and vcfdist (v2.5.3) for the evaluation. Benchmark was downloaded from `https://ftp-trace.ncbi.nlm.nih.gov/ReferenceSamples/giab/data/AshkenazimTrio/analysis/NIST_HG002_DraftBenchmark_defrabbV0.015-20240215/` with the reference GRCh38. NG50: breaks regions on new phase block. Switch NGC50: breaks regions on new phase block or switch error. switchflip NGC50: breaks regions on new phase block, switch error or flip error.

Supplementary Table S5: Number of Hi-C mate-pairs used for phasing

datasets	FALCON-Phase			Diphase		
	correct	incorrect	accuracy (%)	correct	incorrect	accuracy (%)
HG00733	202,594	154,398	56.75	744,172	337,153	68.82
NA12878	236,847	183,979	56.28	707,504	386,692	64.66
HG002	127,727	78,432	61.96	659,916	219,348	75.05
HG002 simplx	169,385	31,501	84.32	1,484,816	189,150	88.70
HG002 duplex	134,397	15,080	89.91	1,242,744	103,713	92.30

Supplementary Table S6: Heterozygous variants affected by switch correction

dataset	assembly	SNP		INDEL		SV	
		with switch correction	no switch correction	with switch correction	no switch correction	with switch correction	no switch correction
HG002	PECAT	2,248,391	2,248,344	408,874	408,875	18,872	18,872
	Shasta	1,906,617	1,906,617	346,918	346,918	15,247	15,247
simplx	PECAT	2,208,683	2,208,681	531,764	531,767	18,276	18,276
	Shasta	2,081,476	2,081,476	419,274	419,274	16,276	16,276
duplex	PECAT	2,210,818	2,210,818	541,861	541,861	18,513	18,513
	Shasta	2,223,701	2,223,701	450,900	450,900	18,270	18,270

Supplementary Table S7: Performance of Diphase using multiple alignments

dataset	method	Genome size (Gb)	N50 (Mb)	Phased block N50 (Mb)	Switch error (%)	Hamming error (%)	Phasing error (%)
HG002	single	3.059	32.8	1.2	3.283100	3.931710	0.982686
		3.061	32.8	1.1	3.421570	4.125022	0.832562
	multiple	3.059	32.8	1.2	3.284611	3.937277	0.982686
		3.060	32.8	1.1	3.420820	4.120342	0.878816
simplx	single	3.013	3.4	0.7	1.537100	3.819070	3.218366
		3.014	3.4	0.8	1.560690	3.821336	2.909211
	multiple	3.014	3.4	0.8	1.546313	3.821356	3.387221
		3.014	3.4	0.7	1.552182	3.820317	2.897278
duplex	single	3.099	38.1	6.2	0.248928	0.463322	0.681748
		3.099	38.1	6.7	0.242676	0.396789	0.431566
	multiple	3.100	38.1	6.4	0.247665	0.459235	0.495203
		3.100	38.1	6.5	0.243926	0.400804	0.432633

Supplementary Table S8: Memory and time usage

dataset	method	memory (G)	total time (h)	BWA-MEM (h)	Clair3 (h)	phasing (h)
NA12878	FALCON-Phase	19.11	34.92	23.81		
	Diphase	50.50	56.80	25.86	28.74	2.03
HG00733	FALCON-Phase	19.04	39.82	23.80		
	Diphase	48.12	44.77	17.51	25.64	1.29
HG002	FALCON-Phase	24.36	49.55	11.56		
	Diphase	49.84	38.50	13.27	22.64	1.66
HG002 simplx	FALCON-Phase	22.03	61.55	11.77		
	Diphase	77.40	27.07	12.46	11.94	2.58
HG002 duplex	FALCON-Phase	24.36	39.68	14.32		
	Diphase	47.27	29.83	13.23	14.51	1.80
HG002 simplx#	FALCON-Phase	21.59	20.50	10.72		
	GFAse	22.14	10.30	9.18		1.12
	Diphase	65.20	34.15	11.25	19.24	3.61
HG002 duplex#	FALCON-Phase	23.14	21.62	11.87		
	GFAse	23.53	11.07	10.10		0.97
	Diphase	46.35	31.10	12.42	16.18	2.40

Shasta assemblies.

FALCON-Phase and GFAse don't run Clair3. Time usage of phasing process in FALCON-Phase can not be acquired.

All experiments were conducted on a server equipped with 48 processors (Intel(R) Xeon(R) Gold 6248R CPU @ 3.0GHz, 192G memory). Diphase requires additional memory for effective phasing as it retains extensive information in the memory for detecting switches, which is deemed acceptable. Diphase demonstrates increased processing time when operating on the HG00733 and NA12878 datasets, while exhibiting decreased processing time on the other datasets. Notably, running Clair3 for SNP calling consumes one third of the entire phasing process, while Diphase's phasing module demonstrates an exceptional speed. However, the SNP calling module remains crucial, significantly enhancing the accuracy of the phasing. Additionally, if the assembly is polished, the mapping of raw reads to the assembly, necessary for the SNP calling process, can be derived from the assembly process.

Algorithm 1 place alternate contigs

Require: primary assembly contigs and alternate assembly contigs
Ensure: filtered mapped segments **S**
1: mapping alternate contigs to their associated primary contigs
2: **for** each alternate contig **do**
3: sort mapped segments according to the start position on primary contig
4: $n \leftarrow$ number of mapped segments
5: compute $f(i)$ for $0 \leq i \leq n$ according to Eq. (1)
6: **for** i from 0 to n **do**
7: **if** $f(i)$ equals to the mapped length of segment i **then**
8: $\mathbf{P}[i] \leftarrow 0$
9: **else**
10: $\mathbf{P}[i] \leftarrow \arg\max \{f(j) + |S_i| - gap(j, i)\}$
11: $i \leftarrow$ index of $\max(f(i))$
12: **while** $\mathbf{P}[i] \neq 0$ **do**
13: $\mathbf{S} \leftarrow$ mapped segment i
14: $i \leftarrow \mathbf{P}[i]$
15: filter duplicated segments and contained segments
16: **return S**

The alternate contigs are firstly mapped to their associated primary contigs using minimap2 v2.22 (line 1). The mapped segments of each alternate contig are sorted by the start position on primary contig (line 2 - 3). The maximal alignment length up to the i-th segment $f(i)$ is calculated as follow: $f(i) = \max\{\max_{i>j\geq 1}\{f(j) + |S_i| - gap(j,i)\}, |S_i|\}$ (line 5). $P[i]$ is used to record the optimal predecessor of segment i (line 6 - 10). The best alignment chain is determined using backtracking starting with the segment with maximum $f(i)$ (line 11 - 14). The alternative contigs may map to the same region in the genome. In these cases, the algorithm prioritizes unique alignments and discards any redundant alignments contained by others (line 15).

Algorithm 2 filter Hi-C alignments

Require: Hi-C alignments **H**, SNP positions **P** on primary contigs, SNP positions **A** on alternate contigs, alignment of alternate contigs to primary contigs

Ensure: filtered Hi-C alignments **F**

1: $\mathbf{S} \leftarrow \emptyset$
2: **for** $a \in \mathbf{A}$ **do**
3: $REF_a \leftarrow$ reference base on a
4: $ALT_a \leftarrow$ alternate base on a
5: $p \leftarrow$ mapped position on the primary contig of a
6: **if** p in **P** **then**
7: $REF_p \leftarrow$ reference base on p
8: $ALT_p \leftarrow$ alternate base on p
9: **if** $REF_a == ALT_p$ and $REF_p == ALT_a$ **then**
10: $\mathbf{S} \cup a$
11: $\mathbf{S} \cup p$
12: **for** $h \in \mathbf{H}$ **do**
13: **if** mapping quality of $h.pair1$ and $h.pair2 \geq q$ and edit distance of $h.pair1$ and $h.pair2 \geq e$ or $h.pair1$ covers position(s) in **S** and $h.pair2$ covers position(s) in **S** **then**
14: $\mathbf{F} \cup h$
15: **return F**

Set S is used to store the position of SNPs in primary contigs and alternate contigs (line 1). For each SNP position a on alternate contigs, let REF_a and ALT_a be the REF base and ALT base called by Clair3 on a (line 3 - 4). Let p be the mapped position on primary contig of a (line 5). If a SNP is called by Clair3 (line 6), let REF_p and ALT_p denote REF base and ALT base called by Clair3 on p (line 7 - 8). If REF_a equals to ALT_p and REF_p equals to ALT_a, p and a are put in set S (line 9 - 11). For each Hi-C pair h, if both reads of h satisfies one of the following conditions will be retained: 1) having a mapping quality score greater than a specified threshold, q (default 10), and an edit distance less than a threshold, e (default 5); or 2) covering at least one SNP retained in S.

Algorithm 3 Switch detecting

Require: filtered Hi-C alignments $\mathbf{H}$, pair information $\mathbf{P}$

Ensure: blocks containing switch $\mathbf{B}$

1: calculate coverage $\mathbf{C}$ of each block with $\mathbf{H}$
2: $\mathbf{B} \leftarrow \emptyset$
3: **for** each block b **do**
4:     **if** sharp drop detected in $\mathbf{C}_b$ **then**
5:         $\mathbf{B} \cup b$
6: **for** $b \in \mathbf{B}$ **do**
7:     **if** $\mathbf{P}[b]$ not in $\mathbf{B}$ **then**
8:         remove b from $\mathbf{B}$
9: **return B**

The algorithm first calculates the coverage of each block (line 1). Set B is used to store the block which is detected a switch on it (line 2). If there is a sharp drop in the coverage curve of b, it will be put into the candidate set B (line 3 - 5). A block b in B will be removed, if there is no switch detected on its corresponding haplotype block (line 6 - 8).

Algorithm 4 Phasing

Require: blocks $\mathbf{B}$ to be phased, Hi-C contacts $\mathbf{H}$, pair information $\mathbf{P}$, iteration $iter$

Ensure: phase $\mathbf{R}$ for each block

1: random set the phase of block b, $\mathbf{R}[b]$ to 1 or -1 and set the phase of b's pair $\mathbf{P}[b]$, $\mathbf{R}[\mathbf{P}[b]]$ to $\mathbf{R}[b] * -1$;

2: $\max_a \leftarrow 0$

3: **for** $i = 0$ to $iter$ **do**

4:     $\max_l \leftarrow 0$

5:     calculate local maximal m according to Eq. (4) by randomly changing the phases of block b and b'

6:     **if** $m > \max_l$ **then**

7:         $\max_l \leftarrow m$

8:     **if** $\max_l > \max_a$ **then**

9:         $\max_a \leftarrow \max_l$

10:         $\mathbf{R} \leftarrow$ the phases of the blocks in this iteration

11:     random select a block b, flip the phases of b, b's pair $\mathbf{P}[b]$, and the neighbor of b and $\mathbf{P}[b]$

12: **return R**

1 Randomly set θ_b to be 1 or -1 for a haplotig b, and for its corresponding haplotig b', set $\theta_(a')$ to be the reverse phase (line 1).

2 For a random phase block b and b', change θ_b and $\theta_{b'}$ from 1 to -1 or vice versa if the objective function can be improved (line 4 - 7).

3 Set the new local maximum as the best maximum if it is better than the best maximum so far (line 8 - 10).

4 For the best maximum, change all the neighbors of a phase block. Go to step 2 to look for a new local maximum (line 11).

5 Perform steps 2-4 iteratively for a default of 10,000 times and report the most optimal local maximum (line 3 - 12).

Supplementary Note S1. Phasing error calculation

In an ideal scenario, haplotigs would be devoid of switch errors, with each haplotig exclusively containing either paternal or maternal specific k-mers. Unfortunately, due to limitations in read length, many haplotigs harbor switch errors, some of which adversely impact the evaluation of other metrics. To address this, we introduce a new metric, the ”phasing error rate”, to assess the performance of phasing tools, disregarding the influence of switch errors. The phasing error rate (p) is computed as follows: Let $\theta\left(a_i\right)$ denote the phase of the haplotig a_i, we have

$$\theta\left(a_i\right)=\begin{cases}1, & N_{pat}>N_{mat}\\ -1, & N_{pat}<N_{mat}\end{cases} \tag{1}$$

For the clustering of blocks along each primary contig, the phase of a cluster C_j is determined as

$$\theta\left(C_j\right)=\frac{\sum_{a_i\in C_j}\theta\left(a_i\right)}{\left|\sum_{a_i\in C_j}\theta\left(a_i\right)\right|}\text{ if }\left|\sum_{a_i\in C_j}\theta\left(a_i\right)\right|\neq 0 \tag{2}$$

If $\left|\sum_{a_i\in C_j}\theta\left(a_i\right)\right|=0$, the phase error of cluster C_j will not be calculated as it means that cluster C_j can represent either a paternal or maternal haplotype. This results in the value of $\theta\left(C_j\right)$ being either 1 or -1. A haplotig a_i in cluster C_j is considered as wronglyy phased if $\theta\left(a_i\right)*\theta\left(C_j\right)=-1$. Thus, the phase error for each cluster C_j is calculated as

$$E\left(C_j\right)=\sum_{a_i\in C_j}\left(abs(\theta\left(a_i\right))\middle|\ \theta\left(a_i\right)*\theta\left(C_j\right)=-1\right) \tag{3}$$

The overall phasing error rate is determined by evaluating all the blocks using the equation

$$p=\frac{1}{N}\sum_{C_j}E\left(C_j\right) \tag{4}$$

where N is the total number of blocks. Substituting Equation (3) into Equation (4), we obtain

$$p=\frac{1}{N}\sum_{C_j}\sum_{a_i\in C_j}\left(abs(\theta\left(a_i\right))|\theta\left(a_i\right)*\theta\left(C_j\right)=-1\right) \tag{5}$$

Supplementary Note S2. Phase shasta assemblies

Shasta has the capability to generate a phased representation where each phased and unphased region is output separately, convertible to a primary/alternate assembly format. For our analysis, we utilized Shasta to assemble two R10 datasets. Using a Python script, we transformed the "Assemble-Phased.fasta" file output by Shasta into a primary/alternate assembly format to execute FALCON-Phase and Diphase. According to the "Naming schemes for assembled segments and paths" of Shasta, We connect phased regions (haplotype 0) and unphased regions belonging to the same bubble chain to one primary contig according to their position. The phased regions with haplotype 1 are treated as alternate contigs. Subsequently, we compared the performance of FALCON-Phase, GFAse, and Diphase. We executed GFAse using the default parameters for Shasta with additional argument "–use_simple_chainer". The contigs unphased by GFAse are output into an individual file "unphased.fasta" which are excluded from analysis. Due to the Shasta assemblies on Nanopore R9 datasets exhibit poor phasing, we refrained from evaluating the performance of both Diphase, GFAse, and FALCON-Phase on those datasets. As shown in Supplementary Table S3, the switch error and Hamming error of alternate contigs in the HG002 dataset are considerably higher than those in the other two R10 datasets, which can impact the performance of the phasing tools.

Supplementary Note S3. Commands for assembly

Command for PECAT

PECAT (v0.0.2 or v0.0.3) was run to assemble genomes. PECAT was run to assemble simplx and duplex reads. PECAT was run with the command "pecat.pl unzip cfg" for assembling. Parameters for NA12878:

```
project= human
reads= $READ
genome_size= 3000000000
threads=$THREADS
cleanup=1
grid=local
prep_min_length=3000
prep_output_coverage=80
corr_iterate_number=1
corr_block_size=4000000000
corr_filter_options=--filter0=l=5000:al=2500:alr=0.5:aal=8000:oh
    =3000:ohr=0.3
corr_correct_options=--score=weight:lc=10 --aligner edlib:bs
    =1000:mc=6 --min_coverage 4 --filter1 oh=1000:ohr=0.01 --
    candidate n=600:f=30 --min_identity 70 --min_local_identity
    60
corr_rd2rd_options=-x ava-ont -f 0.005 -I 10G
corr_output_coverage=80
align_block_size=12000000000
align_rd2rd_options=-X -g3000 -w30 -k19 -m100 -r500 -I 10G -f
    0.002
align_filter_options=--filter0=l=5000:aal=6000:aalr=0.5:oh=3000:
    ohr=0.3 --task=extend --filter1=oh=300:ohr=0.03
asm1_assemble_options=--max_trivial_length 10000

phase_method=2
phase_rd2ctg_options=-x map-ont -c -p 0.5 -r 1000
phase_use_reads=1
phase_phase_options= --coverage lc=30 --phase_options icr=0.1:
    icc=8:sc=10
phase_filter_options = --threshold 1000
```

```
phase_clair3_command = singularity exec -B `pwd -P`:`pwd -P`
   clair3_v0.1-r12.sif /opt/bin/run_clair3.sh
phase_clair3_options=--platform=ont --model_path=/opt/models/
   r941_prom_sup_g5014  --include_all_ctgs
phase_clair3_rd2ctg_options=-x map-ont -c -p 0.5 -r 1000
phase_clair3_phase_options= --coverage lc=15 --phase_options icr
   =0.1:icc=4:sc=10 --filter i=70
phase_clair3_use_reads=0
phase_clair3_filter_options= --threshold=2500 --rate 0.05

asm2_assemble_options=--reducer0 "best:cmp=3,0.1,0.1|phase:sc=3"
    --contig_format dual,prialt

polish_map_options = -x map-ont -w10 -k19 -I 10g
polish_use_reads=0
polish_filter_options=--filter0 oh=2000:ohr=0.2:aalr=0.5

polish_cns_options =

polish_medaka = 1
polish_medaka_command = singularity exec -B `pwd -P`:`pwd -P`
   medaka_v1.7.2.sif medaka
polish_medaka_map_options = -x map-ont  -w10 -k19 -I 10g
polish_medaka_cns_options = --model r941_min_hac_g507
```

Parameters for HG00733

```
project= human
reads= $READ
genome_size= 3000000000
threads=$THREADS
cleanup=1
compress=0
grid=local
prep_min_length=3000
prep_output_coverage=80
corr_iterate_number=1
corr_block_size=4000000000
```

```
corr_filter_options=--filter0=l=5000:al=2500:alr=0.5:aal=8000:oh
    =3000:ohr=0.3
corr_correct_options=--score=weight:lc=10 --aligner edlib:bs
    =1000:mc=6  --min_coverage 4 --filter1 oh=1000:ohr=0.01 --
    candidate n=600:f=30 --min_identity 70 --min_local_identity
    60
corr_rd2rd_options=-x ava-ont -f 0.005 -I 10G
corr_output_coverage=80
align_block_size=12000000000
align_rd2rd_options=-X -g3000 -w30 -k19 -m100 -r500 -I 10G -f
    0.002
align_filter_options=--filter0=l=5000:aal=6000:aalr=0.5:oh=3000:
    ohr=0.3 --task=extend --filter1=oh=300:ohr=0.03
asm1_assemble_options=--max_trivial_length 10000

phase_method=2
phase_rd2ctg_options=-x map-ont -c -p 0.5 -r 1000
phase_use_reads=1
phase_phase_options= --coverage lc=30 --phase_options icr=0.1:
    icc=8:sc=10
phase_filter_options = --threshold 1000

phase_clair3_command = singularity exec -B ‘pwd -P‘:‘pwd -P‘
    clair3_v0.1-r12.sif /opt/bin/run_clair3.sh
phase_clair3_options=--platform=ont --model_path=/opt/models/
    r941_prom_hac_g360+g422  --include_all_ctgs
phase_clair3_rd2ctg_options=-x map-ont -c -p 0.5 -r 1000
phase_clair3_phase_options= --coverage lc=15 --phase_options icr
    =0.1:icc=4:sc=10 --filter i=70
phase_clair3_use_reads=0
phase_clair3_filter_options= --threshold=2500 --rate 0.05

asm2_assemble_options=--reducer0 "best:cmp=3,0.1,0.1|phase:sc=3"
     --contig_format dual,prialt

polish_map_options = -x map-ont -w10 -k19 -I 10g
polish_use_reads=0
polish_filter_options=--filter0 oh=2000:ohr=0.2:aalr=0.5
```

```
polish_cns_options =

polish_medaka = 1
polish_medaka_command = singularity exec -B ‘pwd -P‘:‘pwd -P‘
   medaka_v1.7.2.sif medaka
polish_medaka_map_options = -x map-ont -I 10g
polish_medaka_cns_options = --model r941_prom_high_g4011
```

Parameters for HG002

```
project= human
reads= $READ
hic_reads= ../data/hic_1.fastq.gz;../data/hic_2.fastq.gz
genome_size= 3000000000
threads=$THREADS
cleanup=1
grid=local
prep_min_length=3000
prep_output_coverage=80
corr_iterate_number=1
corr_block_size=4000000000
corr_filter_options=--filter0=l=5000:al=2500:alr=0.5:aal=8000:oh
   =3000:ohr=0.3
corr_correct_options=--score=weight:lc=10 --aligner edlib --
   filter1 oh=1000:ohr=0.01 --candidate n=600:f=30
corr_rd2rd_options=-x ava-ont -f 0.005 -I 10G
corr_output_coverage=80

align_block_size=12000000000
align_rd2rd_options=-X -g3000 -w30 -k19 -m100 -r500 -I 10G -f
   0.002
align_filter_options=--filter0=l=5000:aal=6000:aalr=0.5:oh=3000:
   ohr=0.3 --task=extend --filter1=oh=300:ohr=0.03
asm1_assemble_options=--max_trivial_length 10000

phase_method=2
phase_rd2ctg_options=-x map-ont -c -p 0.5 -r 1000
phase_use_reads=1
```

```
phase_phase_options= --coverage lc=30 --phase_options icr=0.1:icc
   =8:sc=10
phase_filter_options = --threshold 1000

phase_clair3_use_reads=0
phase_clair3_command = singularity exec -B ‘pwd -P‘:‘pwd -P‘
   clair3_v0.1-r12.sif /opt/bin/run_clair3.sh
phase_clair3_options=--platform=ont --model_path=/opt/models/
   ont_guppy5/ --include_all_ctgs
phase_clair3_rd2ctg_options=-x map-ont -c -p 0.5 -r 1000
phase_clair3_phase_options= --coverage lc=30 --phase_options icr
   =0.1:icc=3:sc=10 --filter i=70
phase_clair3_filter_options = --threshold 2500 --rate 0.05

asm2_assemble_options=--reducer0 "best:cmp=2,0.1,0.1|phase:sc=3"
   --contig_format dual,prialt --min_identity 0.98

polish_map_options = -x map-ont -w10 -k19 -I 10g
polish_use_reads=0
polish_filter_options=--filter0 oh=2000:ohr=0.2:aalr=0.5
polish_cns_options =

polish_medaka = 1
polish_medaka_command = singularity exec -B ‘pwd -P‘:‘pwd -P‘
   medaka_v1.7.2.sif medaka
polish_medaka_map_options = -x map-ont -w10 -k19
polish_medaka_cns_options = --model r941_prom_sup_g507
```

Parameters for simplx

```
  project= human
reads= $READ
genome_size= 3000000000
threads=$THREADS
cleanup=0
compress=0
grid=auto
prep_min_length=3000
prep_output_coverage=80
```

```
corr_iterate_number=1
corr_block_size=4000000000
corr_filter_options=--filter0=l=5000:al=2500:alr=0.5:aal=8000:oh
   =3000:ohr=0.3
corr_correct_options=--score=weight:lc=10 --aligner edlib:bs=1000:
   mc=6  --min_coverage 4 --filter1 oh=1000:ohr=0.01 --candidate n
   =600:f=30 --min_identity 90 --min_local_identity 80
corr_rd2rd_options=-x ava-ont -f 0.005 -I 10G -K10G
corr_output_coverage=60
align_block_size=12000000000
align_rd2rd_options=-X -g3000 -w30 -k19 -m100 -r500 -I 10G -f
   0.002 -K 10G
align_filter_options=--filter0=l=5000:aal=6000:aalr=0.5:oh=3000:
   ohr=0.3 --task=realign --filter1=oh=300:ohr=0.03:i=95
asm1_assemble_options=--max_trivial_length 10000

phase_method=2
phase_rd2ctg_options=-x map-ont -w 10 -k19  -c -p 0.5 -r 1000 -I
   10G -K 8G
phase_use_reads=1
phase_phase_options= --coverage lc=20 --phase_options icr=0.1:icc
   =8:sc=10
phase_filter_options = --threshold 1000

phase_clair3_command = singularity exec -B ‘pwd -P‘:‘pwd -P‘
   clair3_v0.1-r12.sif /opt/bin/run_clair3.sh
phase_clair3_rd2ctg_options=-x map-ont -w10 -k19 -c -p 0.5 -r 1000
    -I 10G -K 8G
phase_clair3_use_reads=0
phase_clair3_phase_options= --coverage lc=20 --phase_options icr
   =0.1:icc=3:sc=10 --filter i=90
phase_clair3_filter_options = --threshold 2500 --rate 0.05
phase_clair3_options=--platform=ont --model_path=/opt/models/
   r941_prom_sup_g5014  --include_all_ctgs

asm2_assemble_options=--reducer0 "best:cmp=2,0.1,0.1|phase:sc=3"
   --contig_format prialt,dual
```

```
polish_map_options = -x map-ont -w10 -k19 -I 10g -K 8G -a
polish_cns_options =
polish_use_reads=0
polish_filter_options=--filter0 oh=2000:ohr=0.2:i=96

polish_medaka = 1
polish_medaka_command = singularity exec -B `pwd -P`:`pwd -P`
   medaka_v1.7.2.sif medaka
polish_medaka_map_options = -x map-ont -w10 -k19 -I 10g -K 8G
polish_medaka_cns_options = --model r1041_e82_260bps_sup_g632
polish_medaka_filter_options=--filter0 oh=2000:ohr=0.2:i=96
```

Parameters for duplex

```
  project= human
reads= $READS
genome_size= $GNOME_SIZE
threads=40
cleanup=0
grid=auto
prep_min_length=3000
prep_output_coverage=60
corr_iterate_number=1
corr_block_size=4000000000
corr_correct_options=--score=weight:lc=8 --aligner diff:s=500 --
   min_coverage 1 --filter1 oh=1000:ohr=0.01 --min_identity 95 --
   min_local_identity 90 --candidate n=600:f=30
corr_filter_options=--filter0=l=5000:al=2500:alr=0.5:aal=5000:oh
   =3000:ohr=0.3
corr_rd2rd_options=-X -g3000 -w30 -k19 -m100 -r500 -f 0.002 -K8G -
   I8G
corr_output_coverage=60
align_block_size=4000000000
align_rd2rd_options=-X -g3000 -w30 -k19 -m100 -r500 -f 0.002 -K8G
   -I8G
align_filter_options=--filter0=l=5000:aal=6000:aalr=0.5:oh=3000:
   ohr=0.3 --task=extend --filter1=oh=50:ohr=0.01 --aligner diff:s
   =100 --min_identity 0.90
asm1_assemble_options= --min_identity 0.99 --min_coverage 1
```

```
phase_method=2
phase_rd2ctg_options=-x map-ont -w10 -k19 -c -p 0.5 -r 1000 -I 10G
phase_use_reads=1
phase_phase_options= --coverage lc=8 --phase_options icr=0.02:icc
   =3:sc=4 --filter=i=95.00:alr=0.80:oh=100:ohr=0.01:ilid=100

phase_clair3_command=singularity exec --containall -B ‘pwd -P‘:‘
   pwd -P‘ -B /tmp:/tmp clair3_v0.1-r12.sif /opt/bin/run_clair3.sh
phase_clair3_use_reads=0
phase_clair3_options=--platform=ont --model_path=/opt/models/
   ont_guppy5/ --include_all_ctgs
phase_clair3_rd2ctg_options=-x map-ont -w10 -k19 -c -p 0.5 -r 1000
    -I10G -K 8G
phase_clair3_phase_options=--coverage lc=8 --phase_options icr
   =0.02:icc=2:sc=4 --filter i=95
phase_clair3_filter_options=--threshold=2500 --rate 0.05
asm2_assemble_options= --reducer0 "best:cmp=2,0.1,0.1|phase:sc=2"
   --min_identity 0.99 --max_trivial_length 10000 --contig_format
   dual,prialt --min_coverage 1

polish_map_options=-x map-ont -w10 -k19 -I 10G -K 8G -a
polish_filter_options=--filter0 oh=1000:ohr=0.1:i=98
polish_cns_options=
polish_medaka=1
polish_medaka_command= singularity exec --containall -B ‘pwd -P‘:‘
   pwd -P‘ medaka_v1.7.2.sif medaka
polish_medaka_map_options=-x map-ont -w10 -k19 -I 10G -K 8G
polish_medaka_cns_options = --model r1041_e82_400bps_sup_g615
polish_medaka_filter_options=--filter0 oh=1000:ohr=0.1:i=98
```

Commands for Shasta

Shasta (v0.11.1) was run to assemble simplx and duplex reads. Parameters for simplx

```
shasta --Assembly.mode2.phasing.minLogP 20 --config Nanopore-
   Phased-R10-Fast-Nov2022 --input $READ --thread $THREADS
```

Parameters for duplex

```
shasta --config Nanopore-Phased-R10-Slow-Nov2022 --input $READ --
   thread $THREADS
```

The following conmand was used to connect the haplotigs in "Assembly-Phased.fasta" file to generate primary/alternate format contigs.

```
python /path/to/Diphase/script/preprocessing.py shasta --ifname
   Assembly-Phased.fasta --prefix $prefix --min_length 3000
```
